# Unconventional Actions of Glycoprotein Hormone Subunits: A Comprehensive Review

**DOI:** 10.3389/fendo.2021.731966

**Published:** 2021-09-21

**Authors:** Bruno Querat

**Affiliations:** Unité de Biologie Fonctionnelle et Adaptative, UMR8251 CNRS, Université de Paris, Paris, France

**Keywords:** glycoprotein hormone, TSH, hCG, CGA, unconventional action, cystine knot, GPA & GPB, thyrostimulin

## Abstract

The glycoprotein hormones (GPH) are heterodimers composed of a common α subunit and a specific β subunit. They act by activating specific leucine-rich repeat G protein-coupled receptors. However, individual subunits have been shown to elicit responses in cells devoid of the receptor for the dimeric hormones. The α subunit is involved in prolactin production from different tissues. The human chorionic gonadotropin β subunit (βhCG) plays determinant roles in placentation and in cancer development and metastasis. A truncated form of the thyrotropin (TSH) β subunit is also reported to have biological effects. The GPH α- and β subunits are derived from precursor genes (*gpa* and *gpb*, respectively), which are expressed in most invertebrate species and are still represented in vertebrates as GPH subunit paralogs (*gpa2* and *gpb5*, respectively). No specific receptor has been found for the vertebrate GPA2 and GPB5 even if their heterodimeric form is able to activate the TSH receptor in mammals. Interestingly, GPA and GPB are phylogenetically and structurally related to cysteine-knot growth factors (CKGF) and particularly to a group of antagonists that act independently on any receptor. This review article summarizes the observed actions of individual GPH subunits and presents the current hypotheses of how these actions might be induced. New approaches are also proposed in light of the evolutionary relatedness with antagonists of the CKGF family of proteins.

## 1 The Glycoprotein Hormones in Vertebrates: An Overview

### 1.1 Pituitary Glycoprotein Hormones

**Figure 1:**
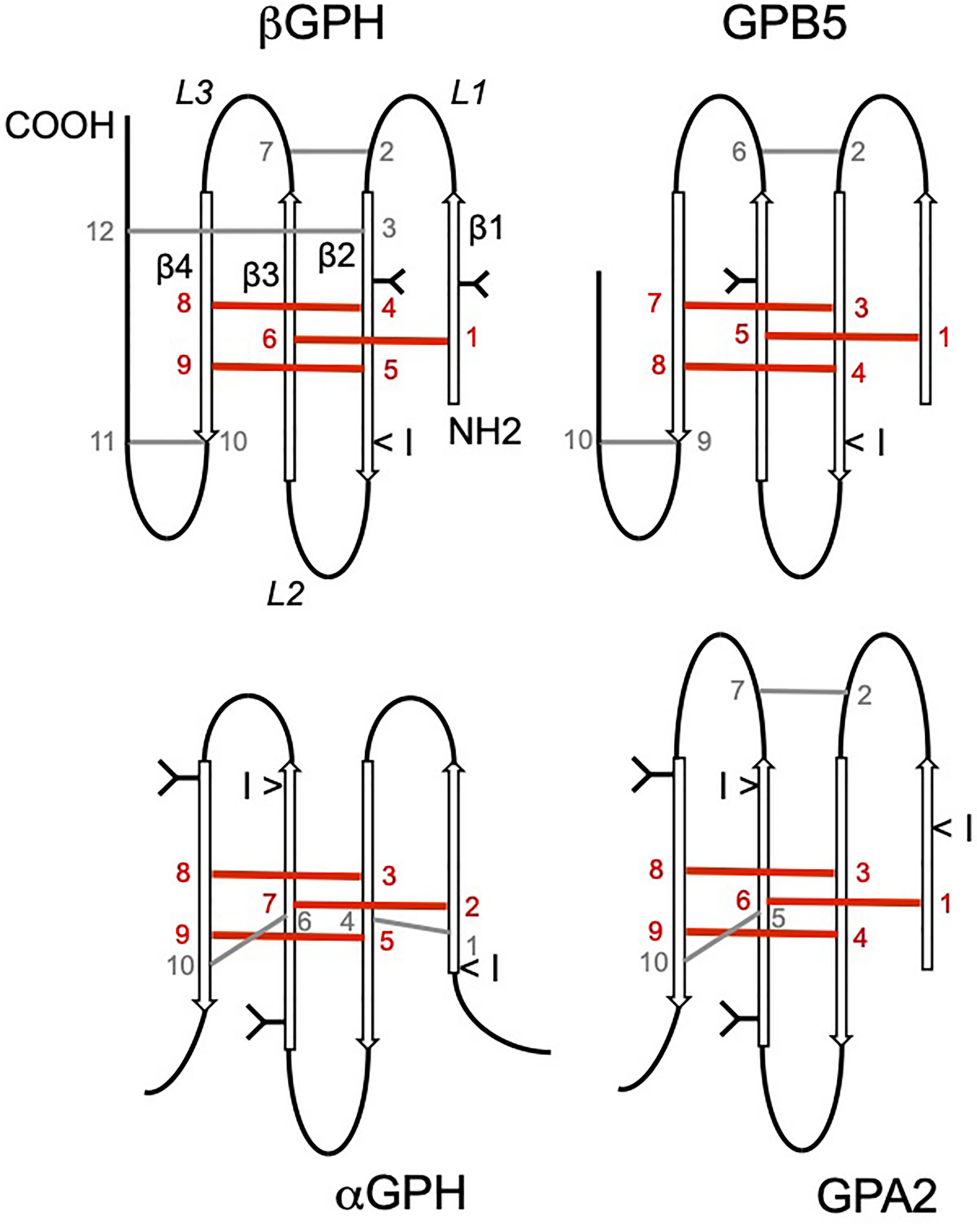
Schematic representation of the glycoprotein hormone (GPH) α- and β subunits and comparison with predicted structure of GPH paralogs GPA2 and GPB5. *N*-linked glycosylated chains are represented by Y shapes: for GPHβ subunits, the first position (from the N-terminal) is common to LH- and FSHβ subunits whereas the second one is common to TSH- and FSHβ subunits. Location of the conserved intron splicing site is indicated by <I symbol. The conserved cysteine residues are numbered. Disulfide bonds are represented by bars. Bars and cysteine numbering are given in red when involved in the cystine-knot structure and in grey for the additional ones. Location of the β strands (β1–β4) and loops (*L1*–*L3*) is indicated.

The glycoprotein hormones (GPH) are heterodimeric glycoproteins composed of an α subunit that is common to all of them and a β subunit that confers the hormonal specificity to the dimer. The GPH family in vertebrates essentially comprises two gonadotropins, the luteinizing hormone (LH) and follicle-stimulating hormone (FSH) and a thyroid-stimulating hormone (TSH) (1–3). The common α- subunit and LHβ, FSHβ, and TSHβ subunits are each encoded by a single gene (*cga*, *lhb*, *fshb* and *tshb*, respectively). Both α and β subunits are glycosylated (**Figure 1**). The α subunit harbors two *N*-glycosylation sites at conserved positions. Two *N*-glycosylation sites are also usually present in FSHβ subunit (only one in most teleost species) (4), whereas LHβ and TSHβ subunits have only one. The position of these glycosylation sites is also conserved between the β subunits, one being common to LHβ and FSHβ subunits and the other one common to TSHβ and FSHβ subunits. From a structural point of view, the GPH subunits belong to the superfamily of cystine-knot proteins and particularly to the transforming growth factor β (TGFβ) subfamily of growth factors (5–7). These cystine-knot proteins are characterized by unique cysteine bonding arrangement that confers a flat and elongated structure fostering their association into homo or heterodimers with or without intermolecular disulfide bonding. Each GPH subunit contains four antiparallel β strands tightened with the core cystine-knot motif from which three hairpin loops are deployed (**Figure 1**). The α and β subunits contain a total of 10 and 12 cysteine residues, respectively, with three pairs of them involved in the cystine-knot motif. GPHs are assembled in a head to tail association so that loops 1 and 3 from one subunit are intertwined with loop 2 of the other subunit. The non-covalently associated GPH heterodimer is stabilized by a « seatbelt » mechanism involving an intramolecular cysteine bridge that closes a carboxy-terminal buckle of the β subunit, encircling loop 2 of the α subunit (8). GPH heterodimers act by activating related but specific receptors (GPHR, namely LHR, FSHR and TSHR) (9–11). Among the large G-protein-coupled receptor superfamily, GPHRs belong to the family of Leucine-rich repeat receptors (LGR type A), characterized by a particularly long extracellular domain that adopts a concave structure to receive the GPH heterodimer with both subunits in contact with the receptor (7, 11). Consequently, none of the GPH subunits is able to satisfactorily (at physiological level) activate any GPHR in a non-heterodimeric association (1). Upon binding, GPHs usually activate the protein Gs-dependent cAMP pathway (12).

The thyrotropin and the two gonadotropins are mainly produced by the pituitary and secreted toward the general circulation (13, 14). TSH is essentially produced by a specific cell-type of the *pars distalis* (PD), the thyrotropes. A second site of synthesis of TSH in birds and mammals is located in the *pars tuberalis* (PT), a structure connected to the mediobasal hypothalamus (15). The two gonadotropins are usually concomitantly but, independently produced by the same gonadotrope cells in mammals. In teleosts, each hormone is synthesized by a specific cell-type (4). In addition to gonadotropes and thyrotropes, the anterior pituitary gland contains adrenocorticotropin hormone-secreting cells or corticotropes, growth hormone-secreting cells or somatotropes, prolactin-secreting cells or lactotropes as well as non-endocrine cells. Among these latter cells are the folliculostellate cells that form a network connecting them to hormone-secreting cells (14, 16).

In the female (most studies have been performed in mammals), FSH is responsible for the maturation of the follicles through its receptor expressed on granulosa cells. LH activates its receptor on theca cells to stimulate steroid synthesis. Some of these steroids are further transformed in the granulosa cells under the influence of FSH during folliculogenesis and of LH later on. The final stage of maturation of oocyte and granulosa cells and ovulation are activated by LH acting through its receptor, which, at this stage, is expressed on granulosa cells. In the male, LH acts in the testis to stimulate steroidogenesis by the Leydig cells whereas FSH acts on Sertoli cells to control spermatogenesis (1, 17). The main action of the TSH from the PD is to stimulate thyrocytes to produce the thyroid hormones (TH) (18). GPH receptors are also expressed in a number of different tissues and GPHs have been found to be involved in a variety of functions by activating these receptors (19–21).

Although submitted to some control, the pituitary GPHα subunit is usually produced in large amount and the dimer formation is dependent on the rate-limiting β subunits. The expression of each GPHβ subunit is under specific regulations (22–24). The hypothalamus provides the main activators of the GPHs production. These stimulators are short peptides produced by specific neurons that are delivered by pulses into the blood that irrigates the anterior pituitary gland. The gonadotropin-releasing hormone (GnRH) preferentially stimulates the production of *lhb* or *fshb* subunit genes according to pulse amplitude and frequency (25). GnRH also stimulates the synthesis of the GPHα subunit in gonadotrope cells, notably when delivered at high pulse frequency (26). The GPHs are tightly regulated by feedback mechanisms arising from their target organs (27). Gonadal steroids exert a sustained retroactive effect on both the hypothalamus and the pituitary gonadotropes to modulate the circulating level of each of the two gonadotropins. Other gonadal (inhibin, activin), metabolic (leptin, fatty acids) or paracrine and autocrine factors contribute to the fine-tuning of LH or FSH production and secretion. Both PD-TSH α and β subunits are stimulated by the thyrotropin-releasing hormone (TRH) in mammals (28, 29) or corticotropin-releasing hormone (CRH) in most non-mammalian vertebrates (30). The expression of PD-TSH is negatively regulated by TH level (22, 31). Numerous paracrine and autocrine factors also participate to achieve an appropriate TH circulating level (32).

### 1.2 GPHs and Their Receptors Are Indispensable for Species Survival

There are no vertebrate species that were shown to lack any of the GPH subunits or GPH receptors, underscoring their irreplaceable role in reproduction or metabolic homeostasis. Inactivation of the *lhb* subunit gene in mice revealed postnatal defects that led to infertility of both sexes with reduced testosterone levels and blocked spermatogenesis in males, low estradiol and progesterone levels and defects in folliculogenesis in females (33). Disruption of the LHR drove to similar phenotypes (34–36). Loss of function of either FSHβ subunit or FSHR generated mice with similar phenotypes, *i.e.* reduced testes volume and altered spermatogenesis but still fertility in males and reduced uterus volume and blocked folliculogenesis leading to infertility in females (37–39). In teleosts, GPHR specificity is not as strict as in mammals and LH can activate the FSHR (4, 40–42) and FSH was also recently shown to activate LHR (43). Consequently, phenotypes of GPH or GPHR-knockout fish are not as deleterious as in mammals. LHβ-null male zebrafish are fertile but females do not spawn (44, 45). FSHβ-deficient zebrafish of both sexes are fertile albeit with delayed gonadal development (45, 46). FSHR knockout male zebrafish are fertile (46) but female zebrafish and medaka displayed arrested growth of follicles at a prepubertal stage (47–49). TSHR-KO mice have non-detectable TH levels, are severely blunted and die after weaning in absence of TH-supplemented diet (50, 51).

### 1.3 Chorionic Gonadotropin

In addition to the two pituitary gonadotropins and TSH that are present in all vertebrate branches, an additional heterodimeric gonadotropin is produced in primates (1) and in equids (52) mainly by the placenta. In primates, the locus encompassing *lhb* has been submitted to serial tandem duplication events giving rise to 6 functional *lhb*-derived genes in human (53–55). Most of them are essentially expressed, together with the GPHα subunit, by syncytiotrophoblastic cells in the placenta to produce the chorionic gonadotropin (CG). In equids, both the *cga* and *lhb* genes have acquired the ability to be expressed by specialized placental cells to produce the so-called LH-CG. In human, CG (hCG) appears to be also produced at low level by the pituitary (56–59). The sequence of these duplicated CGβ-encoding genes (*cgb)* in a given primate species is well conserved between each other and with *lhb*, thanks to a conservative mechanism of gene conversion that uses parts of *lhb* as the template (53). The CGβ subunits however differ from the parent LHβ subunit by an extended carboxy-terminal end that results from a single nucleotide deletion allowing the bypass of the stop codon and the incorporation of the entire 3’ untranslated region of the parent *lhb* (60). The equine LH-CGβ subunit also has a serine-rich carboxy-terminal extension resulting from an independent but similar in effect, frameshift mutation (52). The serine-reach extension allows the attachment of several *O*-linked glycosylated chains in addition to the *N*-linked sugar chains harbored by both the GPHα and LHβ or CGβ subunits. It is to be noted also that hCGβ subunit has an additional *N*-linked glycosylation chain relative to hLHβ subunit providing two carbohydrate chains at the same position as in the FSHβ subunit. These characteristics dramatically increase the half-life of hCG compared to LH. Recently, a new splicing variant of the *cga* has been characterized in human and other primates (61). This variant includes a 31 amino acid-long Alu-J-type segment few amino acids downstream of the signal peptide. It is reported to be expressed exclusively in the placenta within the same cells as the CGβ subunit and to confer an even longer half-life in the circulation to the dimer it forms with hCGβ subunit. Both equine LH-CG and primate CGs exclusively bind to the LHR, which in these species is called LH-CGR. The equine LH-CG (eLH-CG also called pregnant mare serum gonadotropin or PMSG) is able to bind to mammalian FSHR but only in non-equine species (9, 62). The principal actions of hCG have been extensively reviewed (63–65). Among them and through the LH-CGR, hCG has been demonstrated to stimulate progesterone production by the *corpus luteum*, promote angiogenesis for the development of the uterine vasculature, stimulate the differentiation of cytotrophoblast cells, and promote quiescence of the uterine *myometrium*. hCG also contributes to the reduction of the immune response towards the fetus (66).

## 2 Glycoprotein Hormone Related Proteins

### 2.1 GPH-Related Proteins in Vertebrates: A Phylogenetic and Structural Relatedness

The GPH α and β subunits are derived from two precursor genes present since the emergence of bilaterians (2, 67, 68). A locus encompassing these precursor genes is assumed to have tandemly duplicated just before the radiation of vertebrates to generate the ancestral GPHα- and β subunit encoding genes (2). The vertebrate forms of these precursor genes where first characterized in human (67) so that they were named *gpa2* or *gpha2*, *i.e.* GPH α subunit type 2-encoding gene (to take into account the existence of the already known and classical GPH α subunit) and *gpb5* or *gphb5, i.e.* GPHβ subunit type 5 encoding gene (LHβ, FSHβ, TSHβ and CGβ subunits counting for types 1-4). They share numerous structural characteristics with the GPH subunits (67). They have a very similar cysteine residue arrangement including the cystine-knot motif (**Figure 1**). GPA2 bears two *N*-linked carbohydrate chains, one of which in a conserved position with the GPHα subunit (69). GPB5 has only one *N*-glycosylated site, located on a different position as in GPHβ subunits. The main differences concern the carboxy-terminal end of GPB5 that is much shorter than in GPHβ subunits and the absence of the two cysteine residues involved in the so called « seatbelt » mechanism that is crucial for the dimer to be kept tighten once diluted in the general circulation (70, 71). As for the classical GPHs, GPA2 and GPB5 can nevertheless combine into a heterodimer. However, neither human GPA2 was shown to stably associate with any of the human GPHβ subunits, nor GPB5 with the GPHα subunit (72). Of note however, a recombinant heterodimer was produced by combining the lamprey unique (up to now) gonadotropin β subunit with lamprey GPA2. This exotic heterodimer, when expressed as a single chain, was able to activate one of the two identified lamprey GPHRs *in vitro* (73). No specific receptor has been found yet for the GPA2/GPB5 heterodimer in vertebrates. Nonetheless, the recombinant mammalian GPA2/GPB5 heterodimer is several times more potent than TSH in activating homologous TSHR (69, 72), but does not activate homologous LHR and FSHR. The GPA2/GPB5 heterodimer was consequently named thyrostimulin (72). As for the GPH subunits, GPA2 and GPB5 do not activate TSHR as monomers at physiological level. To date, the role of GPA2 and GPB5 in vertebrates has mostly been investigated according to the thyrostimulin concept, *i.e.* as a heterodimer activating the TSHR (69, 74–80). No obvious or conserved role has been found or confirmed for the GPA2/GPB5 heterodimer to date. In addition, if the GPA2 knock out has not been described yet, mice lacking GPB5 were healthy with no deleterious phenotype (69, 74, 77).

### 2.2 GPH-Related Precursor Proteins Outside Vertebrates

The genome of most invertebrate groups contains genes encoding the precursor forms of the αGPH/GPA2 and βGPH/GPB5 subunits (67, 68, 81, 82) that will thereafter be referred to as *gpa* and *gpb*, respectively. Although GPHs LH, FSH and TSH are restricted to vertebrates, a *gphr* related (LGR-1, a type A LGR) gene was identified in several non-vertebrate groups (81, 83, 84) that arose with the emergence of bilaterians (68). The recombinant fly GPA/GPB heterodimer (but not individual subunits) binds to and activates the expressed fly LGR-1 (85), confirming a functional relationship between GPHRs and LGR-1s. In agreement with a functional relationship between GPA (*flr-2*), GPB (*flr-5*) and LGR-1 (*flr-6*) in the nematode *Caenorhabditis*, they all constitute together with another uncharacterized gene (*flr-7*), one of the classes of genes involved in resistance to fluoride ions (86). In the fly, ablation of GPB5-expressing cells reduced the survival rate, demonstrating a determinant role of this protein during larval development (87). However, as for vertebrates, no conserved role or role that will be shared in several unrelated species has been put forward to date for the invertebrate GPA/GPB heterodimer (83).

## 3 Unconventional Actions of GPHs or Their Subunits

A number of observations made in vertebrates shows that some of the GPH subunits are produced and released as monomers or homo-multimers and may induce cellular responses independently of GPHR activation and even in cells that do not express GPH receptors. This review article will summarize results obtained for the individual GPH subunits (**Table 1**), and will also question possible actions for individual GPA and GPB in invertebrates and for their vertebrate derivatives. TSH also presents structural variants that behave in unconventional ways that will be briefly presented in this review.

**Table 1:** Individual subunits sites of synthesis and action.

GPH subunit	Site of synthesis	Known activity	Related section
αGPH	Pituitary cells	lactotrope differentiation in rat	3.1.1
		stimulation of prolactin release from late developing ovine pituitary	
		stimulation of prolactin release from bullfrog pituitary	
	Endometrium	stimulation of prolactin release from decidual cells in human	
	Myometrium	stimulation of decidualization in synergy with progesterone in human stimulation of prolactin synthesis and secretion	
	Prostate	unknown	
	Breast cancer	unknown	3.1.2
βhCG	Extravillous trophoblast	trophoblast migration and invasion into decidua	3.2.1
		stimulation of IL8 production by peripheral blood mononuclear cells	
		proliferation of uterine natural killer cells	
	Cancer cells	proliferation of cancer cells and promotion of metastasis	3.2.2
	Testis	unknown	

### 3.1 Occurrence and Actions of the Free GPHα Subunit

#### 3.1.1 GPHα Subunit and Lactotrope Differentiation and Prolactin Production

During the ontogeny of the pituitary gland in mice and human, the GPHα subunit production starts long before that of any GPHβ subunits (88–91). This free GPHα subunit is released from human fetal pituitary tissue in culture in large amount, exceeding by 200 times the amount of LH and FSH (92). In human fetuses, the circulating level of the α subunit peaks at ten times the level of heterodimeric glycoprotein hormones (93–96). Even in adults, the α subunit is released as a non heterodimeric form (97–100), circulating in the blood in parallel with LH during the menstrual cycle (101, 102) and increasing according to GnRH stimulation in women (97) as well as in men (103). The free α subunit differs from that associated in GPH heterodimers by presenting additional saccharide branching resulting in a slightly heavier molecular mass (104, 105). Homodimers of the α subunit were characterized in both β subunit producing and non-producing cells (106). The α subunits are more tightly associated in the α homodimer than in the heterodimers (106). While the synthesis of large excess of α subunit allows the regulation of the heterodimer production to rely on the rate-limiting β subunit, the released free, heavily glycosylated α subunit may also serve other purposes and possibly, play a role by itself.

Purified α subunit was indeed shown to be involved in stimulating lactotrope differentiation in rat fetal pituitary explants. This action was prevented by the addition of an antiserum against the α subunit but not by an antiserum against the LHβ subunit (107). The α subunit was further demonstrated to have trophic effects on lactotrope cells expansion and production of prolactin on 14-day-old rat pituitary cells allowed to re-aggregate after dispersal (108). In support for a local paracrine action of the GPHα subunit on lactotropes, a preferential connection between gonadotropes and lactotrope cells has been evidenced in the rat (109–111). A physiological role of the α subunit on lactotrope differentiation or proliferation is also supported by the abundance of lactotrope cells in the pituitary gland of anencephalic human fetuses that lack GnRH innervation (112) and present high amount of GPHα subunit (113). Conversely, mice inactivated for the α subunit gene are almost devoid of lactotrope cells (114, 115). The α subunit also specifically stimulated the release of prolactin in late developing ovine fetal pituitary explants, but only at a time when lactotrope cells proliferate (116), suggesting that in ovine, the α subunit does not accelerate lactotrope differentiation but increases prolactin production from fully differentiated cells. In hamster, a correlation was found between an increase in α subunit expression and prolactin expression after a prolonged short photoperiod exposure (117). Outside mammals, the α subunit was also shown (albeit at relative high doses) to stimulate prolactin release in adult bullfrog pituitary cells in culture (118). It thus seems that the GPHα subunit may endorse a role on lactotrope differentiation and/or in stimulating pituitary prolactin production at least in some tetrapods. It must then act on lactotrope cells by activating or antagonizing a signaling pathway that should be conserved in tetrapods. In teleost fish, effect of the GPHα subunit on prolactin production has not been looked for to my knowledge and lactotropes appear much earlier than β subunit-expressing gonadotropes and thyrotropes during pituitary ontogenesis (119). Consequently, the GPHα subunit is unlikely to play a role on lactotrope differentiation in teleosts except if, similarly as in mammals, it is produced long before the GPHβ subunits in some sort of progenitor cells, which have still to be evidenced.

The pituitary gland is not the only tissue that synthesizes prolactin (120, 121). This is also the case for the human decidual cells of the *endometrium* notably during pregnancy (122). The α subunit is also produced in excess (relative to the hCGβ subunit) by trophoblast cells of the placenta and is secreted in free form all along pregnancy (121, 123–125). Despite the fact that the decidual and pituitary lactotrope cells make use a different prolactin gene promoter (126), purified α subunit was shown to stimulate the release of prolactin from decidual cells isolated from term placenta at doses within the range of physiological level (127). Intact hCG was also able to elicit a response but at doses high enough for a contamination with dissociated or free α subunit to be responsible for the effect. Decidualization of endometrial stromal cells also occurs during the luteal phase of the menstrual cycle (126) and, remarkably, these decidualizing cells are able to dissociate hCG into its subunits (128). The free or dissociated α subunit acts synergistically with progesterone to stimulate the transdifferentiation of dispersed stromal cells in culture into decidual cells as monitored by morphological change and the amount of prolactin release (128, 129). The isolated α subunit was twenty times more potent than the intact hCG, with a dose-dependent, saturable and biphasic stimulation pattern (128). *Myometrium* also appears to be one of the multiple extrapituitary sites of prolactin production and the α subunit was demonstrated to stimulate the synthesis and secretion of prolactin from explant cultures of *myometrium* tissue either from pre- or post-menopausal women at equimolar concentration as intact hCG (130). It is then established that the GPH α subunit by itself is able to enhance prolactin release in different mammalian cell types in which the prolactin gene expression is controlled by different promoters. Excessive free α subunit serum level was linked to molar pregnancies (124) even if this alteration might as well be a consequence rather than a cause for this disorder.

#### 3.1.2 GPH α Subunit in Men

In men, in addition to the pituitary, the α subunit was shown to be produced by fibroblasts in the prostate stroma (131) and in rare epithelial cells presenting neurite-like extensions organizing a network toward the lumen of the *acini* (132). These cells do not produce any GPHβ subunit. The free α subunit is released into the seminal fluid at extremely high levels similar to those reached in extra-embryonic coelomic fluid of pregnant women and is present mostly as monomer but also as αα homodimer or even tetramer (132). Seminal level of the free α subunit was shown to be lower in men presenting semen abnormalities, suggesting it may play a role in the latest stages of spermatogenesis (133). Also, the GPH α subunit reduced the growth of stromal smooth muscle cells and it was speculated that the decreasing production of the α subunit along ageing could favor transdifferentiation of fibroblasts into smooth muscle cells and the development of benign prostate hyperplasia (131).

#### 3.1.3 GPH α Subunit in Cancer

Very high GPHα-subunit expression (up to thousand times its level in normal breast tissue) is detected in more than 30% of breast tumors (134). The GPHα-subunit expression is limited to tumor cells positive for the estrogen receptor α for which it could be a reliable marker of functionality. Histopathological observation of α subunit-expressing cells suggested that it could also be, together with progesterone and estrogen-receptor expression, a marker of low tumor aggressiveness (134). However, the role of the α subunit in breast cancer development has still to be unveiled.

### 3.2 Occurrence and Unconventional Actions of the CGβ Subunit

Unconventional, paracrine actions of hCG have been reported for more than twenty years and were recently reviewed (63, 135–137) and only effects that are not suspected to be dependent on a LH-CGR activation or to result from possible contaminants present in the preparations used will be mentioned below.

#### 3.2.1 CGβ Subunit and Pregnancy

In addition to the heterodimeric CG, the human placenta also releases, although in much lower amount, each of its α and β subunits in free form (138). While the GPHα-subunit serum level increases continuously during pregnancy, both hCG and the free hCGβ subunit peak at 8-10 weeks of gestation (125). The ratio between βhCG and dimeric hCG was highest at the beginning of pregnancy, decreasing until 12-13 weeks when it was maintained at about 0.5% (123). In human, the placenta is characterized as hemochorial with the trophoblast entering deep inside the uterus *myometrium* and establishing direct contact with the maternal blood. The primitive *syncytium* is the first cell type that presents invasive behavior (139). The early mononucleated villous cytotrophoblast (VCT) cells are derived from the trophoectoderm and form an epithelium that covers the floating villi. They then fuse into multinucleated syncytiotrophoblast (ST) that will be renewed all along pregnancy. Later on, the highly proliferating extravillous trophoblast (eVCT) cells develop at the tip of anchoring chorionic villi, forming multi-layered columns of cells. As they further differentiate, eVCT cells break away from the column and invade *decidua* whereas cells in the column differentiate into giant multinucleated cells that spread to cover the embryonic sac (139–141). hCG is produced in these two different types, ST and eVCT, of trophoblast cells. Interestingly, the eVCT cells were shown to produce a hyperglycosylated form of hCG, hCG-H (142, 143). An antibody was raised, antibody B152, that selectively recognizes hCG-H but not regular hCG (144, 145). hCG-H represents the major if not the only form of hCG that circulates in the mother serum at the beginning of the first trimester of gestation (146). It is questioned whether the eVCT cells are the first cells to produce hCG-H as the primitive *syncytium* already has an invasive behavior and hCG-H is the major form expressed at that time of implantation (135).

Trophoblast invasion, as measured by migration of eVCT-derived HIPEC65 cells, was stimulated by eVCT-conditioned medium but not by ST-condition medium containing the same amount of hCG. The stimulatory effect was strongly decreased when hCG was depleted from eVCT-conditioned medium using an anti-hCG antibody able to recognize either or not hyperglycosylated forms of hCG (147). Since regular hCG from ST did not stimulate HIPEC65 cell migration, hCG-H from eVCT was unlikely to act through the LH-CGR (136). Moreover, hCG-H was shown to activate LH-CGR with a much lower potency than hCG (138, 148). The importance of hCG-H on trophoblast cell invasion was more recently confirmed. Firstly, treatment of first trimester placental villous tips cultured on collagen droplets with the antibody B152 significantly decreased outgrowth area. Secondly, invasion of JEG-3 choriocarcinoma cells (that produce hCG-H, see below) through a gold electrode in a Boyden chamber assay was similarly inhibited when co-incubated with the B152 antibody (149). In a different approach using JEG-3 cell migration through a basement membrane as an invasion assay, both hCG and hCG-H as well as, most importantly, their purified β subunits were shown to stimulate cell invasion (150). The isolated β subunits were shown to be unable to efficiently activate LH-CGR (148), giving strength to a mechanism of cell invasion independent on LH-CGR expression. Importantly, LH-CGR knockdown of JEG-3 cells did not alter the stimulatory effect of hCG and its β subunit on cell invasiveness. Together, these results demonstrate that hCG-H as well as the β subunit of hCG and hCG-H are able to stimulate trophoblast invasion through an LH-CGR-independent pathway. For these functions, hCG-H action can be reproduced by the β subunit alone, showing that the α subunit is not involved in its action.

Gestation implies a modification of the immune response in the mother to tolerate the blastocyst implantation. hCG was proposed to be a player in this mechanism by stimulating interleukin IL-8 production by peripheral blood mononuclear cells (151). The effect, which was significant only with high doses of recombinant hCG (more than 10 UI/ml), was prevented when the preparation was pre-incubated with an anti-hCG antibody. Intact hCG at high doses (more than 1 µg/ml) but not deglycosylated hCG nor LH or FSH, stimulated the proliferation of uterine natural killer (uNK) cells (152). The verified absence of LH-CGR expression in these two latter cell types (and the high doses required for the effect to be observed) suggested that, here again, LH-CGR-independent mechanisms had to be accounted for.

In agreement with all these LH-CGR-independent actions, LHR knocked-out mice transplanted with pieces of wild type ovary at a prepubertal stage were able to become pregnant, showing that extragonadal LHR expression was not necessary (153). Similarly in human, expression of LH-CGR in uterus or other mother tissues was shown to be dispensable for successful pregnancy. A patient with biallelic inactivating mutations of LH-CGR became pregnant using donor eggs (the actual protocol was not given) and delivered a healthy child, showing that successful implantation and termed pregnancy could be achieved in absence of conventional action of regular hCG in non-placental tissues (154). Only the embryonic tissues were able to express a functional receptor for conventional actions of hCG in this patient. On the other hand, clinical investigation demonstrated that a proportion of hCG-H relative to regular hCG under 50% on the day of implantation in urine (155) or 11 days after blastocyst transfer in serum (156) was predictive for pregnancy failures. Specifically, low hCG-H proportion during the first trimester of gestation was associated with preeclampsia, which is characterized by an impaired invasion of the cytotrophoblast into the *myometrium* (157). Since hCG-H is the major form of hCG produced at the beginning of pregnancy, these results are consistent with a determinant role of hCG-H specifically in the first steps of placental development, a role that is demonstrated to rely on a LH-CGR-independent mechanism. An elegant hypothesis was developed that draws a link between the successive mutations that allowed the hCGβ subunit to increase the number of its *O*-glycosylated residues and its biopotency during primate evolution and the depth of implantation leading to hemochorial placentation in human (63).

#### 3.2.2 CGβ Subunit in Males

If the GPHα subunit is synthesized by the prostate gland and released as a free form in the seminal plasma, the β subunit of hCG is produced, presumably by Leydig cells, in the *interstitium* of the testis (132). The β-hCG is secreted as a free form and found at high level in the seminal plasma (132, 158, 159). The role of the free β-hCG has not been elucidated but a positive correlation was made between its level in semen and sperm count or total motile sperm density (158, 159).

#### 3.2.3 CGβ Subunit and Cancer

Results reported above show that the β subunit of hCG and, particularly its hyperglycosylated form, has the capacity to stimulate cell migration and invasion and to alter immune responses. These are properties that are also necessary for cancer cell dispersion and metastasis. More specifically, the eVCT cells that form the anchoring villi share numerous similarities with cells harboring tumorigenic phenotype and the further differentiated invading eVCT cells behave more so as to metastatic cells with the notable difference that their invasive capacities are tightly regulated (140). Choriocarcinoma (including JEG-3 cells) and germ cell malignancies have been shown to produce the hyperglycosylated form of hCG, hCG-H (160). Many other cancers, notably epithelial tumors, produce the β subunit, but not the α subunit of hCG, with variable proportion of the hyperglycosylated form over the regular form (161–163). Interestingly, the β subunits of hCG and hCG-H by themselves but not regular hCG were shown to be as potent as hCG-H in promoting cell proliferation on choriocarcinoma, germ and non-germ cancer cell lines that produce at least one of these hCG forms. It clearly indicated that the effect did not require activation of LH-CGR (163). Also, purified β-hCG dose-dependently increased cell number of different bladder carcinoma cell lines, an effect that appeared to result from a reduced apoptosis rate (164). Inversely, inhibition of growth was observed in bladder cancer cell lines that produce β-hCG when an anti-β-hCG antibody was added to the culture medium (165). Similarly, knockdown by shRNA of β-hCG expression in a bladder cancer cell line resulted in significant growth inhibition (166). Also, enhanced apoptosis was obtained in uterine cervical carcinoma cells by silencing their β-hCG production (167). The involvement of β-hCG and specifically its hyperglycosylated form on tumor growth was further demonstrated using a xenograft model with JEG-3 cells transplanted into nude mice. When these transplanted mice were administered the antibody B152, oncostasis occurred whereas tumor increased in size in control mice (168).

In addition to its inhibitory effect on apoptosis, which allows multiplication of cancer cells, β-hCG expression level was positively correlated with metastatic behavior of human cancer cells transplanted into nude mice (169). It was further demonstrated that either β-hCG overexpression or incubation with purified β-hCG was able to alter the shape of two different prostate carcinoma cell lines, reduce cell adhesion and lower E-cadherin expression, a recognized suppressor of tumor invasion and metastasis (170) and stimulate their migratory and invasive capabilities (171). Pro-metastatic roles were also attributed to β-hCG in several other cancer cell lines (172–174). BRCA1 (for Breast Cancer 1) was shown to down regulate β-hCG expression in breast cancer cells so that cancer cells harboring inactivating mutation in BRCA1 have high level of β-hCG (175). In agreement with a role of β-hCG in promoting metastasis, knockdown of β-hCG in BRCA1 mutant HCC1937 cells by siRNA significantly reduced their migration and invasion capacities (175). There was no mention of the α subunit expression in this later study as in too many of the studies using supplementation, overexpression or knockdown of the β subunit. But the α subunit expression is linked to estrogen receptor α in breast tumors (134), which is absent in HCC1937 cells so that the effect cannot be attributed to the dimeric CG.

Overall, it is now admitted that β-hCG expression in cancer tissues and elevated β-hCG serum level are poor prognosis for cancer patients (176). Experiments aiming at lowering the level of circulating hCG as a reversible anti-fertility vaccine had been realized by coupling a composite CG consisting of an ovine α subunit and hCGβ subunit coupled to a toxoid to boost the immune response (177, 178). While the vaccine was highly effective in women exhibiting anti-hCG antibody, only 60-80% of women receiving the treatment generated satisfying amount of antibody (179). This however prompted experiments intended to evaluate the use of immunotherapy against β-hCG to treat β-hCG-expressing tumors. In a phase II trial, patients with colorectal cancer were treated with the specific carboxy-terminal peptide of β-hCG conjugated with diphtheria toxoid to enhance immunogenicity. Vaccination induced anti-hCG antibodies in 72% of patients and those who developed anti-hCG antibody levels above the median value had a significantly better survival rate (45 weeks *vs* 24 weeks) than those with levels below the median value (180). Different strategies have since been explored to use anti-β-hCG vaccination to treat β-hCG expressing cancers that aim at further enhancing the immunogenicity of the vaccine (180–190). None seemed to reach the safety or efficacy level needed to be included as a therapeutic tool.

### 3.3 Unconventional Actions of TSH and Its β Subunit

#### 3.3.1 TSH From the Pars Tuberalis

As mentioned earlier, in addition to the TSH synthesized in the PD, another form of TSH is produced by cells in the PT (15). While PD-TSH is stimulated by hypothalamic TRH and submitted to a TH direct feed-back action as mentioned earlier, TSH expressing cells of the PT are devoid of TRH and TH receptors (191). PT-TSH synthesis is instead triggered by melatonin in mammals (192). The released TSH is then delivered in a retrograde pathway to activate TSHR located in the ependymal layer of the infundibular recess to participate in the regulation of reproductive function, metabolism and immune responses according to changes in day length (15, 193, 194). PT-TSH is also constitutively secreted into the general circulation but, surprisingly, this TSH has low biological activity (195). It was shown that PT-TSH binds to serum immunoglobulin IgG2b and albumin to form an extremely stable macro-TSH complex that protects it from binding to TSHR in the thyroid and reduces its degradation rate. The different behavior of the two TSHs was demonstrated to result from differences in their glycosylation status with the presence of tri-antennary and tetra-antennary *N*-glycans associated with sialic acid specifically on PT-TSH (195). In agreement, patients with primary hypothyroidism (who lack PD-TSH) were shown to secrete a highly sialylated TSH with low bioactivity (196). The PT-TSH extracted from the PT is able to activate the TSHR *in vitro* and it is the complex that it forms in the general circulation that prevents it from activating the receptor. The unconventional action in this case relies on the fact that this TSH does not have endocrine capabilities. Its retrograde pathway has not been deciphered yet (193) but it is highly unlikely that PT-TSH reaches the mediobasal hypothalamus *via* the blood circulation.

#### 3.3.2 A Truncated TSHβ Subunit

A decade ago was discovered a splice variant of the *tshb* gene in mice (197) and human (198). This variant is made of the 3’ end of the last intron of *tshb* that now encodes a nine-amino acid-long stretch that presents features in agreement with a signal peptide (199) and the last exon of *tsb*, generating an N-terminally truncated β subunit. This protein lacks amino acids encoded by the first encoding exon and notably one each of the three paired-cysteine residues involved in the cystine-knot plus one that normally buckles the carboxyl end « seatbelt » of the β subunit around the α subunit (**Figure 1**). In addition, it lacks the unique glycosylation site conserved in TSHβ subunits. The cystine knot is a key feature for the folding and conformation of the GPH subunits. In addition, the carbohydrate side chain has been shown to be involved in storage and secretion of the dimer, in receptor binding and activation and in metabolic clearance of GPHs (7). It seems thus very unlikely that this truncated variant may associate with an α subunit to generate a dimer able to activate the TSHR. Yet, molecular dynamics simulation of monomeric TSHβ subunits in complex with the extracellular domain of the TSHR seems to indicate that the full-length β subunit and the truncated peptide dock in a rather similar way (200, 201). And accordingly, both forms were shown to elicit a response as recombinant monomers on cells expressing the TSHR (197, 201, 202). But for the TSHβ variant to activate the receptor, it needs to be secreted and that was not shown for the recombinant variant peptide as a monomer (201). Release of the TSHβ variant was reported from naturally expressing cells or transfected cells but the released amount appeared to be very low. It was shown that TSHβ variant is expressed in pituitary (at very low level compared with full length βTSH), in splenic and peripheral blood leucocytes, bone marrow hematopoietic cells and bone marrow-derived macrophages (197, 198, 202–204). Bone marrow cells were shown to express very little amount of the GPHα subunit (198) if any (202). Even if some role has been attributed to the truncated variant from macrophages in osteoprotection (202), in immune response with the variant expressed in leucocytes homing to the thyroid after systemic bacterial infection (199, 203) and in the pathology of Hashimoto’s Thyroiditis patients (205), the way this variant may achieve these functions remains to be deciphered. It has to be reminded that the TSHβ variant consists of part of the last intron directly attached to the last exon of the *tshb*. So that the PCR-amplified product from cDNA cannot be distinguished from that obtained from genomic DNA. The mRNA preparation must then be exempt of genomic DNA contamination. In addition, no specific antiserum was raised against the variant so that the immunologically detected protein using anti-TSH antibody (in cell sorting or immuno-histochemistry, for example) also recognizes genuine TSH. It will then be determinant for future studies to at least raise a specific antiserum against this variant. The pituitary cells expressing the TSHβ variant were not identified but as TH treatment induces a decrease in its pituitary expression (200) and as PT thyrotrope cells do not express TH receptors (191), the TSHβ variant is most likely produced by PD thyrotrope cells.

### 3.4 GPA2 and GPB5: Individual Paracrine Factors or Heterodimeric Hormones?

The thyrostimulin concept for the GPA2/GPB5 heterodimer was proposed on the basis of its highly efficient activation of mammalian TSHR (72). However, GPA2/GPB5 heterodimer and TSH appeared to dock to partially overlapping sites on the TSHR and large excess of TSH could not displace the GPA2/GPB5 heterodimer when this later was much more potent than TSH to displace the binding of TSH (69). Mammalian gonadotropins have also been found to potentially activate TSHR even if only the high plasma levels that hCG reaches at the first trimester of pregnancy (or in hCG-expressing cancer patients) have physiological impact on TH production by the thyroid (206). In addition, TSHR is unique among GPHRs in that it is submitted to several post-translational modifications including possible cleavage of a 50 amino acid-long segment of the region linking the extracellular domain to the transmembrane domain, with only 3 disulfide bondings for the extracellular domain to be kept attached to the rest of the receptor (207, 208). The cleavage and possible detachment of the extracellular domain may induce the generation of autoantibodies that can have either stimulating or blocking impact on TSHR activity causing thyroid pathologies like Graves disease (209). It indicates that TSHR, in mammals at least, is more permissive than the other GPHRs to alternative ligand-induced activation. However, in the elephant shark, a cartilaginous fish species, the recombinant GPA2/GPB5 dimer does not bind to homologous TSHR (or any homologous GPHR) expressed on mammalian cells (210). It thus remains to be investigated whether the thyrostimulin concept holds in other groups of vertebrates. In addition, for the TSHR to be activated by the GPA2/GPB5 dimer in mammals, the dimer has to be available. But, likely because of the shorter carboxy-terminal end of GPB5 compared to GPHβ subunits and the absence of the « seatbelt » mechanism (70) the stability of the GPA2/GPB5 heterodimer was demonstrated to be far much labile than for the GPHs (72, 211). Consequently, a GPA2/GPB5 dimer seems more likely to act as an autocrine or paracrine factor than as an endocrine factor, whatever the target receptor may be. Interestingly, the tissue distribution of GPB5 was shown to be far less extended than for GPA2 in human and rat (67, 69, 72, 212, 213). GPA2 and GPB5 may be both expressed in a number of tissues but the pituitary gland was the only tissue in which co-expression of GPA2 and GPB5 has been observed within the same cells to date. However, the cell type involved is not very clear yet: corticotrophs in human (72), unidentified cells but not corticotrophs in rat (69). In addition, the GPA2 mRNA expression level was shown to be up to 100 times higher than that of GPB5 in tissues that expressed the two genes (69, 213). No reliable antiserum has been raised against the GPA2/GPB5 heterodimer or the monomeric glycoproteins allowing quantitation so that the tissue concentrations at the protein level cannot be compared. The difference in tissue distribution however strongly suggests that individual subunits rather than heterodimers may be the real players in most tissues.

GPA2 and GPB5 are both glycosylated in vertebrates and mutation experiments on both glycoproteins have shown that, as for the classical GPHs, the presence of the carbohydrate chains impact on secretion and activation abilities of the dimer (211). In contrast, the number and position of potential *N*-linked glycosylated chains are highly variable in invertebrate GPA and GPB proteins. In most hexapods, either GPA or GPB are devoid of potential *N*-glycosylation site (82). Remarkably, in sea hare or tick, none of the proteins displays a potential site. This questions the ability of these proteins to be secreted. In addition, GPA and GPB may show differential expression patterns, notably during development (82, 214, 215). As mentioned above, fly GPA/GPB heterodimer activates fly LGR-1 to stimulate cAMP production (85). Recent research performed in mosquito demonstrated that recombinant GPA and GPB form heterodimers but only when proteins are attached to each other by a linker segment. Unexpectedly, incubation with crude cellular extracts from cells expressing the tethered proteins led to a switch from a constitutive activity to an inhibition of the mosquito LGR-1 as measured by the cAMP level (216). The conditioned medium had no effect, suggesting the dimer is poorly released or that the biological activity of the secreted dimer is prevented by factors present in the medium. It has to be noted also that a recombinant tethered amphioxus GPA/GPB heterodimer was able to activate the amphioxus LGR-1 receptor (217) but only at very high doses (100 to 1000 ng/ml), hardly compatible with an endocrine effect.

## 4 Proposed Mechanisms of Action for the hCGβ Subunit

Both GPH α subunit and hCGβ subunit are able to induce a cellular response by themselves without the need to be in a heterodimeric form. None of the subunits are able to efficiently activate any GPHR (1, 148) and some of the cellular responses have been obtained on cells that do not express LH-CGR. Both GPHα (105, 106) and hCGβ subunits (218) were shown to form homodimers. The glycosylation pattern of uncombined α subunit differs from that of the dissociated α subunit (105). Whether the free α subunit is acting as a monomer or a homodimer has not been clarified and no mechanism of its action has been proposed yet. In contrast, the monomeric recombinant hCGβ subunit was shown to be as active as the homodimeric ββ-hCG in stimulating bladder cancer cell growth (219). It thus appears that hCG-H and the β subunits of hCG or hCG-H either as monomer or homodimer exhibit activities that are not reproduced by the regular heterodimeric hCG. Obviously, the hyperglycosylated state of the β subunit in hCG-H reveals a structure that is buried inside the regular hCG but seemingly accessible in the free β subunits. And accordingly, the hCG-H-specific antibody B152 recognizes a core-2 *O*-glycan structure on a specific serine residue and surrounding residues in the carboxy-terminal end of β-hCG-H that does not appear accessible in regular heterodimeric hCG (145).

### 4.1 β-hCG-H Alters TGFβ Signaling

The GPH α- and β-subunit structure is characterized by a cystine-knot core motif that has the same general characteristics as in the TGFβ family of peptides (5, 6). This super-family of cystine-knot growth factors (CKGFs) includes five groups of factors in vertebrates : 1- TGFβs together with bone morphogenic factors (BMPs), growth and differentiation factors (GDFs) and activin-inhibin subunits, 2- nerve growth factors (NGF), brain-derived neurotrophic factor (BDNF) and neurotrophic factors 3 and 4 (NTF3, NTF4), 3- platelet-derived growth factors (PDGF), 4- vascular endothelial growth factors (VEGF) and 5- the DAN (differential screening-selected gene aberrant in neuroblastoma) or CAN (Cerberus and Dan) family of BMP antagonists (68). A sixth group of CKGFs, bursicon α and β subunits, has been lost in chordates (see below). Apart from BMP antagonists of the DAN/CAN family, CKGFs play their role through activation of kinase receptor complexes (220). Usually, CKGF dimers bind to a family-specific type-II receptor, a transmembrane receptor with intrinsic serine-threonine kinase activity that, upon binding, recruits and phosphorylates a given type-I receptor of the activin-receptor-like kinase (ALK) type that ultimately phosphorylates one of the SMAD (Similar to Drosophila Mothers Against Decapentaplegic) proteins (220). In addition to the SMAD pathway, TGFβ is also able to act through the extracellular signal-regulated kinase (ERK)/Akt pathway (221). Among these CKGFs, TGFβ is known to be deregulated in many cancers and have dual role during cancer progression. In early stages like in healthy cells, TGFβ promotes cell-cycle arrest and apoptosis and thus plays a tumor-suppressor role. However, in later stages, highly activated TGFβ signaling favors tumorigenesis and metastasis by activating an epithelial-to-mesenchymal transition causing cell evasion and by suppressing immune response (222). These latter properties recall those described for hCG-H or its β subunit and it was suggested that hCG-H or β-hCG could act by interfering with the TGFβ signaling (164). In support, TGFβ type II-receptor was shown to be co-precipitated with an anti-hCGβ subunit antibody from the breast cancer HCC1937 cell line (175). It was shown that overexpression of β-hCG in ovarian cancer cells or addition of purified β-hCG into their culture medium was able to activate ERK independently on LH-CGR presence (223), supporting a possible involvement of TGFβ signalization in β-hCG action. Similar effects of hCG on ERK were demonstrated in various cancer cells (174, 224, 225). However, in many instances, TGFβ-like effects described after exogenous hCG treatments may have been misinterpreted as many preparations of hCG or its β subunit were shown to be contaminated with co-purified TGFβ (226) or with other growth factors like EGF (227), which is also demonstrated to stimulate trophoblast invasion (228). In addition, TGFβ was shown to oppose rather than to mimic the effect of recombinant hCGβ subunit on carcinoma cell apoptosis (164). Moreover, opposite to hCG (149), TGFβ was demonstrated to dose-dependently inhibit the invasive capacity of extravillous trophoblast cells isolated from placental explants at 8-10 weeks of gestation (229, 230). Thus, hCG would better fit an antagonistic role on TGFβ signaling. More work is needed to figure out how hCG-H or the hCGβ subunit may interfere with TGFβ signaling with both similar and opposite effects.

### 4.2 Other Proposed Mechanisms

It was also proposed that hCG could enhance trophoblast invasion by altering the insulin-like growth factor II (IGFII)/mannose 6-phosphate receptor (M6PR) recycling, allowing action of IGFII (231). IGFII is abundantly synthesized by trophoblast cells (232) and was shown to activate trophoblast invasion *via* IGFII/M6PR (233). The mechanism of how hCG could act on receptor recycling was not deciphered. Another again mechanism was proposed for the stimulation of proliferation of uNK cells by hCG (152). It was shown that the hCG action was prevented when deglycosylated hormone was used or when cells were incubated in the presence of excess mannose whose receptor was co-localized with hCG on the cell surface. Similarly, the stimulating effect of hCG on IL8 production by peripheral blood mononuclear cells was attenuated in the presence of excess mannose, suggesting here again that hCG could bind to C-type lectins expressed on cell surface (151). Such a type of interaction would make sense when considering the highly glycosylated form of hCG-H that is expressed at time of implantation and invasion and that is responsible for the LH-CGR-independent action of hCG. However, since the β subunit of regular hCG is equally active as its hyperglycosylated form in promoting cancer cell proliferation (163), other mechanisms have to be looked for. Also, it has to be reminded that for both effects on uNK cell proliferation and peripheral blood mononuclear cells, very high levels of hCG were needed so it has to be demonstrated that such high levels are reached at the site of implantation.

## 5 Phylogenetic Studies Open Up Ways for Alternative Modes of Action for the Individual GPH-Related Proteins

**Figure 2:**
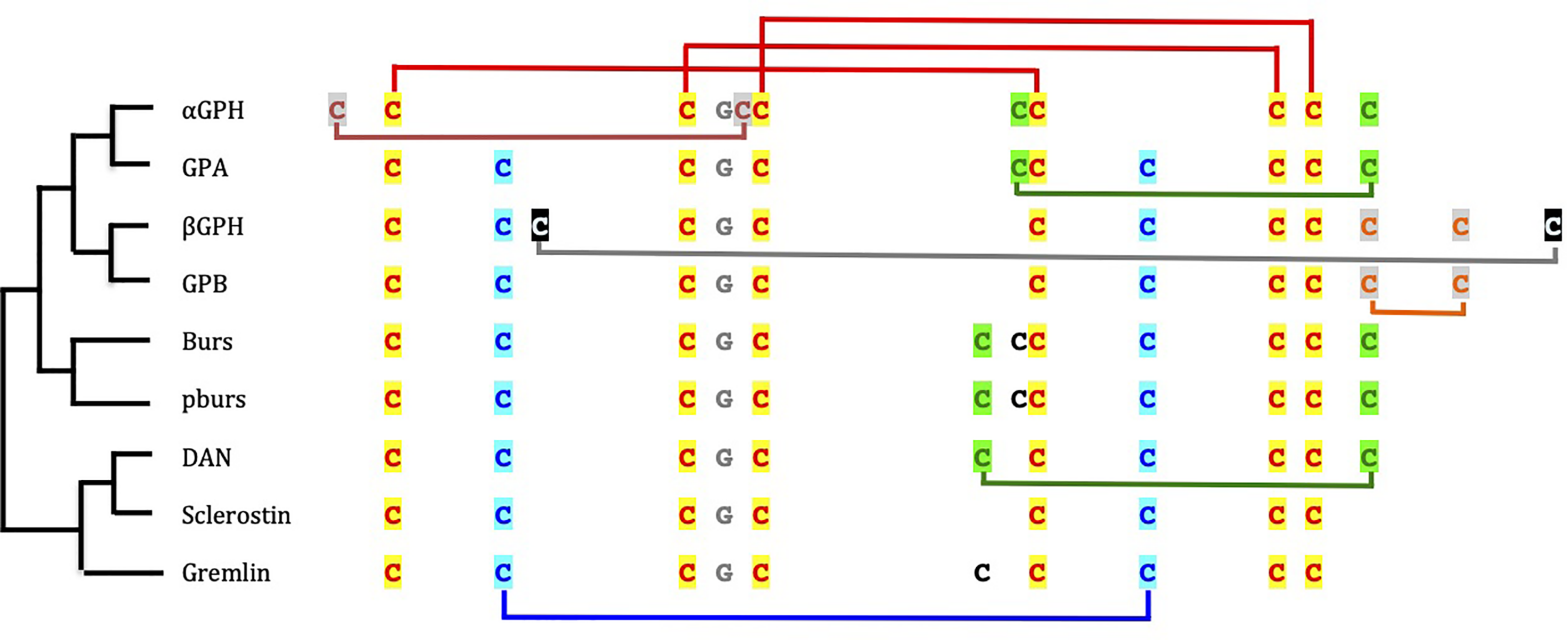
Cysteine residue arrangement of glycoprotein hormone (GPH) subunits and cystine-knot growth factors. The phylogenetic relationships [after (67)] between sequence types are schematically represented on the left side. Disulfide bridges between cysteine residues involved in the cystine-knot are depicted in red on top of the sequences. The other cysteine residues linked by disulfide bridges are given in different colors according to their position in the folded protein. The cysteine residues in black are not involved in intramolecular bridge. The glycine residue conserved in all cystine-knot growth factor is indicated in grey. GPA and GPB are the precursor forms in invertebrates of GPH α- and GPHβ subunits, respectively; Burs is for bursicon α and pburs for bursicon β; DAN is for differential screening-selected gene aberrant in neuroblastoma.

The closest phylogenetically relatives for GPA and GPB (see **Figure 2**) were identified as the CKGF members bursicon, burs (or bursicon α) and pburs (for partner of burs, or bursicon β) (68) that heterodimerize to activate a type-B LGR (LGR-2) (234). Both *bursicon* subunit genes have disappeared from chordate genomes whereas genes for three vertebrate type-B LGR receptors, *lgr4-6* are found (84, 215). In absence of bursicon these receptors were first thought to be orphan in chordates. The three of them were later found to bind with high affinity all four R-spondin members which are potent Wnt enhancers (235). This is an example of molecular exaptation: a receptor has lost its natural ligand but a new ligand comes and uses the receptor. It is to be noted that, in addition to the change in ligand, LGR4-6 also change their signalization as binding of R-spondins on LGRs does not activate protein G-induced signalization (235). Bursicon burs/pburs heterodimer is involved in cuticle melanization and sclerotization after molting and in adult wing expansion in insects (234). Interestingly, bursicon subunits can also associate as burs/burs and pburs/pburs homodimers and both homodimers were shown to stimulate the secretion of several anti-microbial proteins, independently on LGR-2 (236, 237). It thus appears that the ability of homodimers to activate a different signaling system as the heterodimeric ligand does not seem restricted to the GPH-GPHR system.

The next closest phylogenetically relative in line (68) appears to be the DAN-CAN family of BMP antagonists (**Figure 2**). These antagonists were also shown to have the closest structural relationships with the GPH subunits in terms of cysteine residues arrangement (70, 238). This family includes 1- Dan or Nbl1 (for neuroblastoma suppression of tumorigenicity 1), 2- Gremlin, Cerberus, PRDC (protein related to DAN and Cerberus) and Coco/Dante that form a subgroup and 3- SOST (Sclerostin) and USAG-1 (Uterine sensitization-associated gene-1) gathered into another subgroup (239). These proteins are known to be highly expressed and involved during development. Except for SOST and USAG-1, which are active as monomers, DAN family members act as non-covalently linked homodimers. Most of them were shown to bind to selected BMP-GDF members to prevent them from activating their receptor. Gremlin additionally interacts with VEGF receptor 2 whereas SOST and USAG-1 antagonizes Wnt signaling by binding to co-receptors (239, 240). A tentative modeling of their interaction with BMP members has been proposed showing two homodimers of the antagonist binding perpendicularly at each tip of the elongated growth factor (239). So, the secreted peptides of the DAN family of BMP antagonists are able to play their role without activating a specific receptor. Interestingly, some of the DAN family members have been shown to harbor several accessible and effective sulfated polysaccharide heparin and heparan-sulfate binding sites (241). Binding of these factors to heparan-sulfate proteoglycans of the extracellular matrix or to cell surface hampers their diffusion away from the site of secretion and contribute to reaching and maintaining the concentration needed for their action. Whether such sites also exist for the GPH individual subunits and related proteins remain to be explored. Nonetheless, the close structural similarity between GPH-related subunits and these family of antagonists, their ability to form homodimers and their apparent absence of specific receptors open up new exploratory fields for the mechanistic study of their actions. It would be interesting, for example, to search for cystine-knot factors with which GPH subunits could interact and if these interactions could be related to some of their actions.

## 6 Evolutionary Considerations

In invertebrate genomes, the genes encoding GPA and GPB are most commonly found close to each other without intervening gene (68, 82), suggesting that the physical promiscuity between these two genes is under evolutionary pressure. Furthermore, no species has been reported that lacks only either GPA or GPB but some invertebrates, notably among hymenoptera like the parasitic wasp (242), honey and bumble bees (243) and several ants (244), but also the unrelated leech (245) and myriapod (246, 247), appear to lack both GPA and GPB and, interestingly, the LGR-1-type receptor also. The absence of the receptor gene but presence of GPA and GPB genes has not been reported to date. This strongly suggests that the evolutionary conservation of these two proteins and the receptor are interdependent. One would indeed expect that if GPA or GPB had determinant individual LGR-1-independent roles, it should have been conserved even in the absence of its heterodimeric partner or the receptor for the heterodimer. So, it looks like, contrary to what was expected from available functional and expression distribution data, a possible receptor-independent individual role for GPA and GPB would not be the evolutionary drive for their conservation.

**Figure 3:**
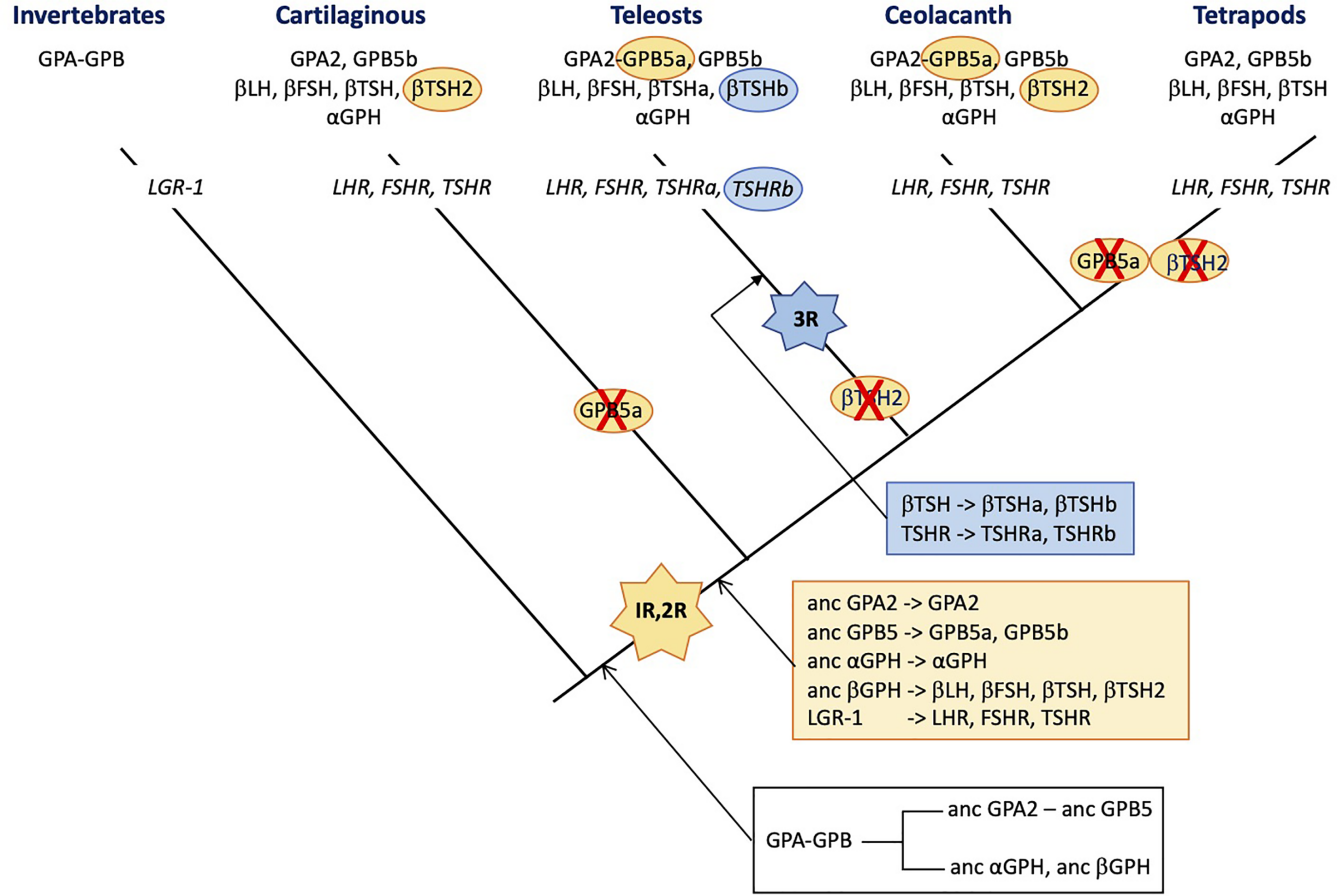
Schematic representation of the distribution of glycoprotein hormone (GPH) subunit-related genes and receptors (in italic) presented on a simplified phylogenetic tree. The initial tandem duplication of GPA and GPB giving rise to the ancestral forms of GPA2 and GPB5 and of α- and β-GPH just prior to the vertebrate expansion is indicated in the black box underneath the tree. The genes issued from the two rounds of genomic duplication that occurred in founder vertebrates are depicted in the orange box. Those issued from the teleost-specific third round of genomic duplication are presented in the blue box. The hyphen in GPA-GPB and GPA2-GPB5a indicates that the genes are organized in tandem. The red cross indicates loss of the gene. 1R, 2R and 3R represent whole genome duplication events. LH, luteinizing hormone; FSH, follicle stimulating hormone; TSH, thyroid stimulating hormone; GPA, invertebrate representative of the precursor for vertebrate GPA2 and GPH α subunit; GPB, invertebrate representative of the precursor for vertebrate GPB5 and GPHβ subunits. GPA2 and GPB5, vertebrate representatives of GPA and GPB, respectively; LGR, leucine-reach repeat receptor; LHR, FSHR and TSHR, receptors for LH, FSH and TSH, respectively.

Interestingly, the tandem organization of GPA and GPB genes observed in most invertebrate species was maintained before the duplication of the locus just prior to the vertebrate emergence as evidenced by comparative synteny analysis between vertebrate and amphioxus (a pre-vertebrate chordate) genomes (2). The two rounds of whole genome duplications (1R and 2R) that occurred before the radiation of vertebrates (248) led to the conservation of only two GPB5-related genes and one GPA2 gene in extent vertebrates (**Figure 3**). One of the two GPB5 genes (GPB5a) was maintained close to the unique GPA2 gene (2, 249), indicating that the evolutionary constraints that maintained this genes in a tight locus in invertebrates were still probably in place in early vertebrates. No additional GPB-related genes were conserved after the third, teleost-specific whole genome duplication (3R). The GPB5a form was lost in tetrapods but is still present, close to GPA2 in coelacanth (which emerged just prior to the tetrapod radiation) and in teleosts (249).

Usually, gene duplicates accumulate detrimental mutations leading to pseudogenization within few million years. Advantageous mutations conferring new functions to the duplicate can exert positive selection allowing the duplicate gene to be maintained for new functions (neofunctionalization) or to take in charge parts of the ancestral gene function (subfunctionalization) (250). There are no vertebrate species lacking any of the GPH subunits or GPH receptors. However, the duplication of the locus comprising GPA and GPB just prior to the vertebrate emergence that generated the precursors of GPA2, GPB5 and GPH α and β subunits, and the two rounds of whole genome duplications (1R and 2R) that occurred in founder vertebrates may have open opportunities for new roles to be acquired by the generated paralogs (250). According to the current hypothesis (2, 3), the ancestral GPHβ subunit generated four paralogs through the 1R and 2R, the β subunits of LH and FSH on the one hand and of TSH and its sister gene TSH2 on the other hand (251) (see **Figure 3**). LH-, FSH- and TSH-β subunits allowed new functions to be acquired by combining to the GPHα subunit as heterodimers and their encoding gene was maintained. The TSH2β subunit sequence is well conserved with only few amino acid replacements and shows the normal cysteine arrangement and unique glycosylation site as compared to the classical βTSH, suggesting it can form heterodimers with the GPH α subunit. This βTSH2-encoding gene was lost in tetrapods and before the radiation of teleosts but is still present in cartilaginous fish, the coelacanth and the dipnoi (another group that emerged just before the terrestrialization). The duplication of *tshβ* was not accompanied by a duplication of the *tshr* (or the duplicated receptor was not maintained), so it is tempting to assume that TSH2 did not acquire a specific role, different from the classical TSH, that would have given a driving force for its evolutionary conservation. In agreement, the elephant shark recombinant TSH2 activated the shark TSHR only when the two subunits were linked to each other (210), questioning the stability of the TSH2β/GPHα heterodimer and the possible role and mode of action of TSH2. No data are yet available for coelacanth or dipnoi TSH2.

Interestingly, after the 3R that occurred just prior to the radiation of teleosts, among the GPHβ subunits, only the duplicated *tshb* gene was maintained. According to the nomenclature used in post 3R teleosts, they are referred to as *tshbb* (encoding βTSHb) and *tshba* (encoding the classical βTSHa). The gene *tsh2b* had disappeared before the radiation of teleosts (249). In the pituitary, the teleost βTSHb appears to be synthesized in a different area as βTSHa, in few cells close to the pituitary stalk, a location that is somewhat similar to the PT in mammals (252). They are submitted to differential regulation of expression and notably, CRH stimulates the TSHa β subunit whereas it has no effect on βTSHb. The similarity with the mammalian PT-TSH is reinforced by the role that TSHb appears to play in the regulation of physiological functions according to the photoperiod (253). TSHb but not TSHa was also shown to be involved in the metamorphic events determinant for the pre-adaptation of salmon to the sea water environment (252) and in the adaptation to different ecosystems in stickleback (254). This time, the 3R-issued duplicated *tshr* gene was also maintained (251) so that teleosts are provided with two TSHR (TSHRa and TSHRb). The new *tshrb* was translocated to a new genomic environment in teleosts soon after its duplication and is thus susceptible to be submitted to new cis-regulatory factors (251). In accordance, the tissue distribution of the novel teleost TSHRb was shown to be much more extended than for the classical TSHRa and notably encompass several brain areas where TSHb is also expressed (251). This pleads for a possible paracrine action of the novel TSHb. It is not known whether both teleost TSH can activate both teleost TSHR. It is to be noted that βTSHb acquired a second glycosylation site, at the same position as in βLH so that βTSHb shares the same two glycosylation site positions as in βFSH. Whether this second glycosylation site would alter the binding specificities of TSHb remains to be explored. The conservation of the duplicated TSH-TSHR system seems to indicate that it has acquired an advantageous function that allowed it to be maintained during evolution of teleosts. It is thus tempting to assume that teleosts and mammals have opted for different ways to divide the different TSH functions (subfunctionalization). They both produce TSH in different cell types allowing differential regulation but in teleosts, a second and possibly specific TSH receptor was used whereas in mammals, the photoperiodic-related PT-TSH was modified so that it could not interfere with the homeostasis-controlling PD-TSH. It would be interesting to look for a possible paracrine role of TSH2 in the regulation of photoperiod or other environment-related adaptations in cartilaginous fish.

Ancestral *gpa2* and *cga* genes are the only genes for which none of the whole genomic duplications either at the root of vertebrates (1R and 2R) or before the radiation of teleosts (3R) generated duplicates that were conserved to extant vertebrates (**Figure 3**). Some species in teleosts do have duplicated genes for the GPH α subunit but phylogenetic analysis showed that they resulted from relatively recent and independent pseudo-tetraploidization events (255). The whole genomic duplications generated five GPHβ subunits (the β subunits of LH, FSH, TSH, TSH2 and of the teleost TSHb). LH, FSH and TSH (and TSHb) acquired specific functions and were maintained (neofunctionalization). It can be hypothesized that TSH2 endorsed only parts of the function (subfunctionalization) of the ancestral TSH issued from the 1R. When this subdivision of the function was not advantageous, the TSH2 β subunit-encoding gene evolved into pseudogenization and disappeared from the genomes of descendant lineages. Given that αGPH has to bind to each of these newly generated β subunits (including β-hCG in primates), one can wonder why the αGPH sister genes have never been conserved. This is all the more surprising as specific functions are now attributed to the individual α subunit. Neo- or subfunctionalization should have represented strong driving forces on the GPH α subunit and would have favored the α GPH duplicates to be maintained. It thus appears that the strongest conservative driving forces would be related to the heterodimerization process which imposes the same constraints on the GPH α subunit whatever the newly duplicated GPHβ subunit in order to generate a heterodimer able to bind and activate a related receptor. The functions that the GPH α subunit may play as monomer or homodimer would not exert strong-enough driving forces for the conservation of a duplicated, neofunctionalizing form. The new CGβ subunit that appeared with the radiation of primates evolved by enriching its glycosylation pattern allowing a deeper implantation and better oxygenation of the fetus blood (63). Here, the neofunctionalization is in progress with the conservation of an increasing number of *CGβ* subunit genes.

## 7 Conclusion

Individual actions have been clearly demonstrated for the GPH α subunit in mammals as well as for the hCGβ subunit in human. The mechanisms of these actions have not been deciphered yet but it is demonstrated that the receptors for the GPH heterodimers are not involved. In invertebrates, GPA, GPB and the GPHR together appear to be under selective evolutionary pressure but individual roles for GPA and GPB can also be predicted. In vertebrates, the thyrostimulin concept of GPA2/GPB5 heterodimer activating the TSHR does not seem to be the driving force for their evolutionary conservation notably as it does not function in a cartilaginous fish. Individual actions are then to be looked for each GPA2 and GPB5 proteins also. Paracrine or autocrine actions rather than endocrine actions are favored for all these factors. Individual functions of the GPH subunits have been shown to be related to modifications of their glycosylation status. For the α subunit specific glycosylation seems to favor homodimerization. The heavily glycosylated chain in hCG exposes specific sites favoring actions *via* the individual β subunit. Also, the PT-TSH-specific glycosylation pattern prevents it from activating the undesired receptor. Close structural relationships have been established between GPH subunits and CKGFs and particularly with members of the DAN family of BMP antagonists that act in a receptor-independent manner. This should stimulate new approaches for future investigations on the mechanisms of receptor-independent actions of individual GPH subunits.

## Author Contributions

The author confirms being the sole contributor of this work and has approved it for publication.

## Conflict of Interest

The author declares that the research was conducted in the absence of any commercial or financial relationships that could be construed as a potential conflict of interest.

## Publisher’s Note

All claims expressed in this article are solely those of the authors and do not necessarily represent those of their affiliated organizations, or those of the publisher, the editors and the reviewers. Any product that may be evaluated in this article, or claim that may be made by its manufacturer, is not guaranteed or endorsed by the publisher.
